# Risk factors of cervical cancer among ethnic minorities in Yunnan Province, China: a case–control study

**DOI:** 10.1097/CEJ.0000000000000704

**Published:** 2021-07-20

**Authors:** Min Zhao, Rong-yan Gu, Song-rui Ding, Lei Luo, Yue Jia, Chen-xin Gao, Bin Chen, Xue-bin Xu, Hong-fen Chen

**Affiliations:** a Medical Administration Department, The Third Affiliated Hospital of Kunming Medical University (Yunnan Cancer Hospital), Xi Shan county; b School of Public Health, Kunming Medical University, Cheng Gong New City; c Department of Gynecology, The Third Affiliated Hospital of Kunming Medical University (Yunnan Cancer Hospital), Xi Shan county, Kunming, Yunnan, China

**Keywords:** case-control study, cervical cancer, risk factors, Ethnic minorities in Yunnan

## Abstract

Cervical cancer is a common malignant tumor of the female reproductive system in the world, which is a serious threat to women’s life and health. According to the latest report, the incidence of cervical cancer is 11.42 per 100 000, and the mortality rate is 3.77 per 100 000 in Yunnan Province, which is still higher than the national average. Although there have been some relevant studies on the risk factors of cervical cancer in recent years, research on ethnic minorities is lacking in Yunnan Province.

**Objective:**

To analyze and explore the related risk factors of cervical cancer in women of ethnic minorities in Yunnan Province, to provide the scientific basis for the development of cervical cancer prevention and control strategies and measures in this region.

**Methods:**

In total 1119 cervical cancer patients diagnosed by histopathology at the Yunnan Cancer Center (Yunnan Cancer Hospital) from January 2010 to December 2019 were selected as the case group. According to the 1:1 matching principle of the case–control study, 1119 patients with nonmalignant tumors of the same nationality, the same hospital, age difference less than 3 years old, were selected as the control group. Univariate and multivariate conditional logistic regression were used for statistical analysis.

**Results:**

Basic medical insurance for rural residents (OR = 3.659; *P = *0.003), human papilloma virus (HPV) infection (OR* = *90.030; *P < *0.001) and concurrent reproductive tract infections (OR = 1.992; *P = *0.047) were risk factors for cervical cancer. Late first marriage(OR* = *0.881; *P = *0.032), the number of normal childbirths ≤2 (OR* = *0.480, *P = *0.033) and contraception (OR* = *0.291; *P = *0.002) were positive factors for cervical cancer.

**Conclusion:**

The high incidence of cervical cancer in Yunnan minority women is the result of many factors: HPV infection is the highest risk factor for cervical cancer, women with reproductive tract infections and basic medical insurance for rural residents have a higher risk for cervical cancer; Late first marriage, the number of deliveries ≤2 and contraception are positive factors for cervical cancer.

## Introduction

Cervical cancer is a common malignant tumor of the female reproductive system in the world, which is a serious threat to women’s life and health (Hanprasertpong *et al* 2017; Zhang *et al* 2020). According to the latest data from the International Agency for Research on Cancer (IARC) of the WHO (https://www.iarc.fr/faq/latest-global-cancer-data-2020-qa/0000), there were 600 000 new cases and 340 000 deaths of cervical cancer worldwide in 2020, ranking the fourth in both incidence and death of female cancer, among them, the number of new cases of cervical cancer in China was 110 000, and the number of deaths was 60 000, the incidence rate was 10.88 per 100 000, and the mortality rate was 3.17 per 100 000. According to the latest report, the incidence of cervical cancer is 11.42 per 100 000, and the mortality rate is 3.77 per 100 000 in Yunnan Province (Hongmei *et al* 2020), which is still higher than the national average, and the age of onset is gradually younger. These make the prevention and control situation of cervical cancer more severe. At the same time, there is the most concentrated area of ethnic minorities in Yunnan Province, China (Chao 2016), mainly including 25 ethnic minorities such as Hui, Yi, Bai, Hani and Dai, which have specific cultures, lifestyles and account for 33.37% of Yunnan’s population. Although there have been some relevant studies on the factors affecting cervical cancer in recent years (Yanhong and Lin 2016), the incidence of cervical cancer is related to a variety of factors, and there are obvious regional differences (Xiaodong 2012; Tanjun 2018), in addition, there is no relevant research on the risk factors of cervical cancer among ethnic minorities in Yunnan Province. Therefore, this study used the case–control study method to analyze and explore the related risk factors of cervical cancer in women of ethnic minorities in Yunnan Province, to provide the scientific basis for the development of cervical cancer prevention and control strategies and measures in this region.

## Objects and methods

### Research subjects

#### Case group

In total 1119 cervical cancer patients diagnosed by histopathology at the Yunnan Cancer Center (Yunnan Cancer Hospital) from January 2010 to December 2019 were selected as the case group.

Case group inclusion criteria:

(1) The cases came from ethnic minorities women in various regions of Yunnan Province.
(2) Newly diagnosed patients with having no previous cervical cancer treatment.
(3) It was confirmed that the diagnosis of cervical cancer was correct.
(4) There were no other malignant tumors.

Case group exclusion criteria:

(1) Patients whose native place were not Yunnan Province.
(2) Patients with malignant tumors in other parts of the body (except for the metastasis of the primary cervical cancer to other parts).

### Control group

According to the 1:1 matching principle of the case–control study, 1119 patients with nonmalignant tumors of the same nationality, the same hospital, age difference less than 3 years old, were selected as the control group.

Control group inclusion criteria:

(1) The control came from ethnic minorities women in various regions of Yunnan Province.
(2) The control and the case were of the same nationality, the same hospital, and age difference less than 3 years..
(3) The result of cervical cytology screening was ‘no malignant cells and intraepithelial lesion cells’.

Control group exclusion criteria:

(1) Patients with severe cervical erosion, cervical precancerous lesions or other malignant tumors.
(2) Cervical cytologic abnormality.
(3) Patients whose native place was not Yunnan Province.

### Research method

Based on the hospital’s information system (HIS) to query the patient’s medical history data and collect the patient’s general demographic data (age, nationality, medical payment method, occupation, marriage, BMI, family history of tumor, etc.), reproductive health history (age of menarche, age of first marriage, age of first pregnancy, times of pregnancy, times of childbirth, times of abortion, history of contraception, history of menopause, etc.), human papilloma virus (HPV) infection, reproductive tract infection and other infections. A case–control study was used to sort out and classify the corresponding indicators of the case group and the control group.

### Statistical analysis

The analyses were performed using IBM SPSS Statistics for Windows, Version 23.0 (IBM Corp, Armonk, New York, USA). The analysis of equilibrium between the case group and the control group adopted the independent sample *t*-test and chi-square test. First, univariate conditional logistic regression was used to analyze the influencing factors of cervical cancer, the variables with statistical significance were screened out, and then multivariate conditional logistic regression was further introduced. The method for independent variables to enter the model was stepwise regression, with the inclusion criterionα being ≤0.05, the exclusion criterionα being ≥0.10 and inspection levelα = 0.05.

## Results

### Equilibrium analysis of case group and control group

There were 1119 patients with cervical cancer in the case group, with an average age of (46.72 ± 9.64) years, and 1119 patients with noncervical cancer in the control group, with an average age of (46.51 ± 9.99) years. There was no statistically significant difference in age between the two groups (*P* > 0.05). There were 25 ethnic minorities in Yunnan Province, including hui, Yi, Bai, Hani and Dai, etc. The case group was completely matched with the control group (*P* = 1.000), and the two groups are comparable. See Table 1.

**Table 1:** Equilibrium analysis of case group and control group

Matching variables	Case, *n* (%)	Control, *n* (%)	*t/x^2^*	*P* value
Age (years)	46.72 ± 9.64	46.51 ± 9.99	0.499	0.618
Ethnic minorities				
Yi	354 (31.60)	354 (31.60)	0.000	1.000
Bai	193 (17.20)	193 (17.20)		
Dai	129 (11.50)	129 (11.50)		
Hani	122 (10.90)	122 (10.90)		
Hui	98 (8.80)	98 (8.80)		
Other	223 (20.00)	223 (20.00)		

### Univariate factor analysis of general demographics and cervical cancer

Medical payment methods were basic medical insurance for rural residents (OR = 2.266; *P < *0.001), full self-finance (OR* = *1.805; *P < *0.001), and the type of occupation were employee (OR* = *2.447; *P = *0.001), self-employed (OR* = *10.959; *P < *0.001), which were risk factors for cervical cancer. Unmarried (OR = 0.240; *P* = 0.006) was a positive factor for cervical cancer. BMI, family history of tumor were not significantly associated with the occurrence of cervical cancer (*P* > 0.05). See Table 2.

**Table 2:** Univariate conditional logistic regression analysis of general demography and cervical cancer

Variables in equations	Case, *n*（*%*）	Control, *n* (%)	*P* value	OR	OR 95% CI
Medical payment method					
Basic medical insurance for urban employees	172 (15.40)	219 (19.60)	0.706	0.953	0.741–1.225
Basic medical insurance for urban residents	179 (16.00)	243 (21.70)	0.396	0.895	0.692–1.157
Basic medical insurance for rural residents	158 (14.10)	88 (7.90)	<0.001	2.266	1.657–3.100
Full self-finance	311 (27.80)	209(18.70)	<0.001	1.805	1.421–2.294
Other types	299 (26.70)	360 (32.20)	–	1.000	–
Occupation type					
Farmer	556 (47.90)	580 (51.80)	0.086	1.498	0.944–2.378
Employee	129 (11.50)	81 (7.20)	0.001	2.447	1.430–4.187
Self-employed	102 (9.10)	15 (1.30)	<0.001	10.959	5.285–22.722
Unemployed	295 (26.40)	390 (34.90)	0.597	1.135	0.709–1.816
Retirement	37 (3.30)	53 (4.70)	–	1.000	–
Marriage					
Unmarried	9 (0.80)	21 (1.90)	0.006	0.240	0.086–0.664
Married	1055 (94.30)	1044 (93.70)	0.116	0.650	0.380–1.112
Widowed	21 (1.90)	27 (2.40)	0.069	0.480	0.217–1.059
Divorced	34 (3.00)	22 (2.00)	–	1.000	–
BMI					
<18.5	94 (8.60)	95 (8.60)	0.985	0.997	0.717–1.387
18.5–24	644 (58.60)	645 (58.40)	0.963	0.996	0.828–1.197
>24	361 (32.80)	365 (33.00)	–	1.000	–
Family history of tumor					
Yes	35 (3.10)	32 (2.90)	0.714	1.094	0.677–1.766
No	1084 (96.90)	1087 (97.10)	–	1.000	–

CI, confidence interval, OR, odds ratio.

“–” means no data.

### Univariate analysis of female reproductive health history and cervical cancer

Late menarche was the risk factor for cervical cancer (OR = 1.075; *P* = 0.002), late first marriage (OR = 0.922; *P* < 0.001), late first pregnancy (OR = 0.922, *P* < 0.001), the number of pregnancies ≤3 (OR = 0.656; *P* < 0.001), the number of normal deliveries ≤2 (OR = 0.493; *P < *0.001), the number of abortions ≤1 (OR = 0.758; *P = *0.003) and contraception (OR = 0.137, *P < *0.001) were the positive factors for cervical cancer. There was no significant correlation between menopause and cervical cancer (*P* *>* .05). See Table 3.

**Table 3:** Univariate conditional logistic regression analysis of female reproductive health history and cervical cancer

Variables in equations	Case, *n* (%)	Control, *n* (%)	*P* value	OR	OR 95% CI
Menarche age (years)	14.63 ± 1.96	14.39 ± 1.92	0.002	1.075	1.027–1.126
First marriage age (years)	21.40 ± 3.30	22.25 ± 3.64	<0.001	0.922	0.897–0.948
First pregnancy age(years)	21.88 ± 3.15	22.67 ± 3.69	<0.001	0.922	0.894–0.951
Number of pregnancies					
≤3	672 (60.10)	760 (68.80)	<0.001	0.656	0.544–0.791
>3	447 (39.10)	345 (31.20)	–	1.000	–
Number of normal deliveries					
≤2	796 (71.10)	893 (81.00)	<0.001	0.493	0.391–0.622
>2	323 (28.90)	210 (19.00)	–	1.000	–
Number of abortions					
≤1	733 (65.50)	788 (71.30)	0.003	0.758	0.631–0.911
>1	386 (34.50)	317 (28.70)	–	1.000	–
Contraception					
Yes	827 (73.90)	1058 (94.80)	<0.001	0.137	0.097–0.192
No	292 (26.10)	58 (5.20)	–	1.000	–
Menopause					
Yes	660 (59.00)	649 (58.00)	0.212	1.333	0.849–2.094
No	459 (41.00)	470 (42.00)	–	1.000	–

CI, confidence interval, OR, odds ratio.

– means no data.

### Univariate factor analysis of HPV and other infections to cervical cancer

HPV infection (OR* = *37.682; *P < *0.001), reproductive tract infections (OR* = *3.273; *P < *0.001) and other infections (OR* = *1.246; *P = *0.008) were risk factors for cervical cancer. See Table 4.

**Table 4:** Univariate conditional logistic regression analysis of HPV and other infection to cervical cancer

Variables in equations	Case, *n* (%)	Control, *n* (%)	*P* value	OR	OR 95% CI
HPV infection					
Yes	950 (84.90)	143 (12.80)	<0.001	37.682	24.675–57.544
No	169 (15.10)	976 (87.20)	–	1.000	–
Reproductive tract infections					
Yes	333 (29.80)	130 (11.70)	<0.001	3.273	2.578–4.155
No	786 (70.20)	985 (88.30)	–	1.000	–
Other infections					
Yes	498 (44.50)	431 (38.70)	0.008	1.246	1.059–1.467
No	621 (55.50)	684 (61.30)	–	1.000	–

Other infections: hepatitis B virus infection, urinary tract infection, HIV infection.

CI, confidence interval, HPV, human papilloma virus; OR, odds ratio.

– means no data.

### Multivariate conditional logistic regression analysis of cervical cancer

Based on univariate conditional logistic regression analysis, 15 variables were finally screened out and introduced into multivariate conditional logistic regression analysis. A total of six variables were statistically significant: basic medical insurance for rural residents (OR* = *3.659; *P = *0.003), HPV infection (OR* = *90.030; *P < *0.001) and concurrent reproductive tract infections (OR = 1.992; *P = *0.047), which were risk factors for cervical cancer. Late first marriage (OR* = *0.881; *P = *0.032), the number of normal childbirths ≤2 (OR* = *0.480; *P = *0.033) and contraception (OR* = *0.291; *P = *0.002), which were positive factors for cervical cancer. See Table 5.

**Table 5:** Multivariate conditional logistic regression analysis of cervical cancer

Variables in equations	Case, *n* (%)	Control, *n* (%)	*P* value	OR	OR 95% CI
Medical payment method					
Basic medical insurance for urban employees	172 (15.40)	219 (19.60)	0.222	0.656	0.334–1.291
Basic medical insurance for urban residents	179 (16.00)	243 (21.70)	0.863	1.071	0.492–2.328
Basic medical insurance for rural residents	158 (14.10)	88 (7.90)	0.003	3.659	1.573–8.515
Full self-finance	311 (27.80)	209 (18.70)	0.192	1.498	0.816–2.748
Other types	299 (26.70)	360 (32.20)	–	1.000	–
First marriage age (years)	21.40 ± 3.30	22.25 ± 3.64	0.032	0.881	0.785–0.989
Number of normal deliveries					
≤2	796 (71.10)	893 (81.00)	0.033	0.480	0.245–0.942
>2	323 (28.90)	210 (19.00)	–	1.000	–
Contraception					
Yes	827 (73.90)	1058 (94.80)	0.002	0.291	0.132–0.641
No	292 (26.10)	58 (5.20)	–	1.000	–
HPV infection					
Yes	950 (84.90)	143 (12.80)	<0.001	90.030	41.313–196.197
No	169 (15.10)	976 (87.20)	–	1.000	–
Reproductive tract infections					
Yes	333 (29.80)	130 (11.70)	0.047	1.992	1.011–3.927
No	786 (70.20)	985 (88.30)	–	1.000	–

CI, confidence interval, HPV, human papilloma virus; OR, odds ratio.

– means no data.

## Discussion

The occurrence and development of cervical cancer is a continuous, complex, multifactorial, multi-step gradual process, with a long reversible period of precancerous lesions (Guolan 2019). Therefore, early detection of risk factors for cervical cancer, systematic and effective screening and early treatment of precancerous lesions are of great significance for the prevention and treatment of cervical cancer (Wen 2005; Xin 2013).

### General demography and cervical cancer

Relevant studies have shown that (Jensen *et al* 2008) occupation, socioeconomic status, and so on were closely related to the incidence of cervical cancer. Bosch *et al.* (1997) pointed out in the study that the difference in the incidence of cervical cancer among women of different types of occupations was statistically significant. In the univariate conditional logistic regression of this study, the risk of cervical cancer is higher for employees and self-employed, but the difference is not statistically significant after multivariate conditional logistic regression analysis. This may be due to the exclusion of confounding factors after multivariate analysis of fixed variables. Previous studies have also shown (Fen 2011) that marital status, such as widowed, divorced, and so on were also risk factors for cervical cancer. In the univariate analysis of this study, unmarried is a positive factor for cervical cancer, but the difference is not statistically significant after the multivariate analysis. Durowade *et al.* (2012) in an analysis of the influencing factors of cervical cancer in Nigerian women showed that family history of the tumor was a risk factor for cervical cancer, but in this study, family history of tumors and BMI are not associated with cervical cancer in ethnic minorities women. In addition, in the univariate and multivariate analysis of this study, it is also found that patients whose medical payment method is basic medical insurance for rural residents (OR* = *3.659) are more likely to suffer from cervical cancer. The reason may be that the majority of patients with basic medical insurance for rural residents may come from remote rural areas, with greater economic pressure and life pressure. Meanwhile, they do not have a strong sense of self-protection and health and neglect the care of their own health due to the influence of specific culture and specific living habits in ethnic minority areas.

### Female reproductive health history and cervical cancer

In the univariate conditional logistic regression analysis of this study, it is found that late first menarche is a risk factor for cervical cancer. Conversely, late first marriage, late first pregnancy, the number of pregnancies ≤3, the number of normal childbirths ≤2, the number of abortions ≤1 and contraception are positive factors for cervical cancer. In the further multivariate logistic regression analysis, it is found that late first marriage (OR* = *0.881), the number of normal childbirth ≤2 times (OR* = *0.480), and contraception (OR* = *0.291) are closely related to cervical cancer, which are the positive factors for cervical cancer. Xin *et al.* (2013) in a case–control study on the risk factors of cervical cancer among rural women in Wuhan found that late first marriage is a positive factor for cervical cancer, which is consistent with the result of this study. The female first marriage age is basically the same as their first sex life age in China. Many studies have confirmed that premature age at first sex life was a risk factor for cervical cancer (Yanfeng *et al* 2011). The female reproductive tract is not mature at the age of 20 years and below. It is easy to cause cervical injury, cervical squamous metaplasia or atypical hyperplasia and increase the risk of cervical cancer if their age of first marriage and first sexual life is too early. In minority areas, women may get married earlier and are more likely to increase the risk of illness due to the influence of ethnic customs. In many studies at home and abroad (Juárez-Cedillo *et al.* 2007; Yanli and Bin 2018), too many times of pregnancy, childbirth and abortion are risk factors for cervical cancer. There is no significant difference in the number of pregnancies and abortions in the multivariate analysis, but the number of childbirth is consistent with the previous research results. Women with too many deliveries will have higher estrogen levels during pregnancy. At the same time, the cervix will be more stimulated and even cause infection if the number of deliveries is too much, which will increase the risk of cervical cancer. Previous studies (Suxia *et al* 2013) have also paid attention to that the use of contraceptive measures, such as the use of condoms, can reduce the occurrence of cervical cancer, which is consistent with the results of this study. The use of contraceptive measures is a positive factor for cervical cancer. However, some studies (De Villiers 2003) suggested that women taking contraceptives increased the risk of cervical cancer, which may be related to the long-term use of contraceptives to increase the level of estrogen and progesterone in the body, but there is no evidence of the relationship between estrogen and progesterone to cervical cancer, so it is controversial whether taking contraceptives is a risk factor for cervical cancer (Xiaodong 2012). In subsequent studies, the impact of different contraceptive methods on cervical cancer can be further analyzed.

### The influence of HPV and other infections to cervical cancer

HPV infection is currently recognized as one of the most important risk factors for cervical cancer (Kamzol *et al* 2012; Rodríguez *et al.* 2012). For example, Li *et al.* (2015) in the Meta-analysis on influencing factors of cervical cancer among Chinese married women found that the OR value of HPV infection was 25.490. In this study, the OR value of HPV infection was as high as 90.030. It shows that HPV infection is more harmful to cervical cancer of ethnic minorities than the general population. In addition, this study also found that reproductive tract infection is also a risk factor for cervical cancer (OR* = *1.992), which may cause various inflammations, such as cervicitis and cervical erosion. The stimulation of inflammation may lead to the replacement of squamous epithelium by columnar epithelium, which increases the risk of cervical cancer. Relevant studies have shown (Caihong *et al* 2011) that the risk of cervical cancer increased with the increase of reproductive tract infection microorganisms. Therefore, early detection, early diagnosis and early treatment of cervical-related inflammation are of great significance to the early prevention of cervical cancer.

### Conclusion

The high incidence of cervical cancer in Yunnan minority women is the result of many factors. It is closely related to the method of medical payment, first marriage age, the number of normal childbirth, contraception, HPV infection and reproductive tract infection. Among them, HPV infection is the highest risk factor for cervical cancer, women with reproductive tract infections and basic medical insurance for rural residents have a higher risk for cervical cancer, late first marriage, the number of deliveries ≤2 and contraception are positive factors for cervical cancer.

## Suggestion

In view of the above influencing factors, it is very important to formulate prevention strategies and measures in line with the cervical cancer of ethnic minority women in Yunnan Province. Such as strengthening the awareness of reproductive health care for women of childbearing age in ethnic minority areas, increasing regular surveys of women of all ages, improving personal hygiene habits and reducing HPV and reproductive tract infection rates. At the same time, advocating late marriage and late childbirth, choosing well-tolerated and effective contraceptive measures, and appropriately reducing the number of deliveries to reduce the stimulation to the cervix, so as to effectively reduce the prevalence and mortality of cervical cancer in this area.

## Acknowledgments

The auyhors are very grateful for the support of The Planning Project of Philosophy and Social Sciences inYunnan Province and technology projects of Yunnan Provincial Archives Bureau.

This study is in line with the requirements of moral ethics. All participants signed informed consent.

The data in this study are true and reliable. The datasets generated and/or analyzed during the current study are not publicly available due because they are related to patients but are available from the corresponding author on request.

This study was supported by grants from The Planning Project of Philosophy and Social Sciences in Yunnan Province (grant number: QN2018022), Science and technology projects of Yunnan Provincial Archives Bureau(2017-y-016).

6.6 Authors’ contributions

M.Z: conceptualization, writing the original draft. R-y.G.: formal analysis, Writing the original draft. S-r.D.: methodology. L.L.: methodology. Y.J.: conceptualization and methodology. C.-x.G., H.-f.C. and B.C.: data curation. X-b.X.: writing the review and editing.

We confirm that all methods were carried out in accordance with the “cervical cancer clinical practice guidelines” from national comprehensive cancer network (NCCN) of the United States.

We confirm that all experimental protocols were approved by Ethics Committee of the Third Affiliated Hospital of Kunming Medical University.

### Conflicts of interest

There are no conflicts of interest.

## References

[R1] Bosch FX Munoz N de Sanjosé S (1997). Human papillomavirus and other risk factors for cervical cancer. Biomed Pharmacother. 51(6–7):268–275. 9309247 10.1016/s0753-3322(97)83542-1

[R2] Caihong L Baohong L Xiaobin L (2011). Distribution characteristics of microorganisms in the vagina of patients with cervical cancer. J China Med Univ. 40:267–271.

[R3] Chao W (2016). A retrospective study of cervical cancer screening results and incidence in Yunnan Cancer Hospital. Kunming Medical University.

[R4] De Villiers EM (2003). Relationship between steroid hormone contra- ceptives and HPV, cervical intraepithelial neoplasia and cervical carcinoma. Int J Cancer. 103:705–708. 12516087 10.1002/ijc.10868

[R5] Durowade KA Osagbemi GK Salaudeen AG Musa OI Akande TM Babatunde OA. (2012). Prevalence and risk factors of cervical cancer among women in an urban community of Kwara State, north central Nigeria. J Prev Med Hyg. 53:213–219. 23469591

[R6] Fen W (2011). Meta analysis of influencing factors of cervical cancer and case-control study on risk factors of cervical cancer in Jiangxi Province. Nanchang University.

[R7] Guolan J (2019). Risk factors of cervical cancer in rural areas of QingYang, Gan Su province: A case-control study. Lanzhou University.

[R8] Hanprasertpong J Geater A Jiamset I Padungkul L Hirunkajonpan P Songhong N (2017). Fear of cancer recurrence and its predictors among cervical cancer survivors. J Gynecol Oncol. 28:e72. 28758378 10.3802/jgo.2017.28.e72 PMC5641523

[R9] Hongmei W Yang C Siying R Jiao G Mingfen Q (2020). Analysis of cancer incidence and mortality in Yunnan cancer registry,2016. J Pract Oncol. 34:485–490.

[R10] https://gco.iarc.fr/today/data/factsheets/cancers/23-Cervix-uteri-fact-sheet.pdf.

[R11] Jensen KE Hannibal CG Nielsen A Jensen A Nøhr B Munk C Kjaer SK (2008). Social inequality and incidence of and survival from cancer of the female genital organs in a population-based study in Denmark, 1994-2003. Eur J Cancer. 44:2003–2017. 18662869 10.1016/j.ejca.2008.06.014

[R12] Juárez-Cedillo T Vallejo M Fragoso JM Hernández-Hernández DM Rodríguez-Pérez JM Sánchez-García S. (2007). The risk of developing cervical cancer in Mexican women is associated to CYP1A1 MspI polymorphism. Eur J Cancer. 43:1590–1595. 17512722 10.1016/j.ejca.2007.03.025

[R13] Kamzol W Jaglarz K Tomaszewski KA Puskulluoglu M Krzemieniecki K (2012). Assessment of knowledge about cervical cancer and its prevention among female students aged 17-26 years. Eur J Obstet Gynecol Reprod Biol. 166:196–203. 23141797 10.1016/j.ejogrb.2012.10.019

[R14] Li L Lingying C Dandan L (2015). Meta–analysis on influencing factors of cervical cancer among Chinese married women. Chin J Hum Sex. 24:42–45.

[R15] Rodríguez AC Schiffman M Herrero R Hildesheim A Bratti C Sherman ME. (2012). Low risk of type-specific carcinogenic HPV re-appearance with subsequent cervical intraepithelial neoplasia grade 2/3. Int J Cancer. 131:1874–1881. 22213126 10.1002/ijc.27418 PMC3356792

[R16] Suxia L Yongxiu Y Huiping Y (2013). Risk factors of cervical cancer in Wudu Gansu China. J Modern Oncology. 21:2793–2795.

[R17] Tanjun L (2018). A case-control study on the risk factors of cervical cancer and precancerous lesions. Chinese Remedies Clinics. 18(S1):69–70.

[R18] Wen C (2005). China’s plans to curb cervical cancer. Lancet Oncol. 6:139–141. 15737830 10.1016/S1470-2045(05)01761-4

[R19] Xiaodong Z (2012). A case-control study on the risk factors of cervical cancer. China Modern Medicine. 19:169–170.

[R20] Xin L (2013). Case-control study on risk factors of cervical cancer in rural women of Wuhan. Huazhong University of science and technology.

[R21] Yanfeng F Jian L Rongxian X (2011). An exploratory study on the related risk factors of cervical cancer and precancerous lesions. J Chin Physician. 13:447–450.

[R22] Yanhong C Lin T (2016). Case control study on risk factors of cervical cancer and precancerous lesions. Chin J Health Statistics. 33:120–122.

[R23] Yanli W Bin W (2018). Research progress on high-risk factors and early screening of cervical cancer. Jilin medicine. **39**:166–169.

[R24] Zhang S Xu H Zhang L Qiao Y (2020). Cervical cancer: epidemiology, risk factors and screening. Chin J Cancer Res. 32:720–728. 33446995 10.21147/j.issn.1000-9604.2020.06.05 PMC7797226

